# Craniofacial fibrous dysplasia: a challenge for general dental practitioners

**DOI:** 10.1038/s41415-025-9010-y

**Published:** 2025-11-28

**Authors:** Manuel W. H. Man, Roshni Ruparelia, Jasleen K. Batra, Krupti Denhard, Ulpee R. Darbar

**Affiliations:** 633450424660957258636 Specialty Doctor in Restorative Dentistry, Royal National ENT and Eastman Dental Hospital, 47–49 Huntley Street, London, WC1E 6DG, United Kingdom; 596280898256360115510 Dental Core Trainee 2 in Restorative Dentistry, Royal National ENT and Eastman Dental Hospital, 47–49 Huntley Street, London, WC1E 6DG, United Kingdom; 086054846780129373312 Dental Core Trainee 1 in Restorative Dentistry, Royal National ENT and Eastman Dental Hospital, 47–49 Huntley Street, London, WC1E 6DG, United Kingdom; 250242553328947602447 Consultant in Restorative Dentistry, Royal National ENT and Eastman Dental Hospital, 47–49 Huntley Street, London, WC1E 6DG, United Kingdom

## Abstract

Craniofacial fibrous dysplasia (CFD) is an asymptomatic disease that can have oral manifestations with severe effects on a patient's wellbeing. It is usually diagnosed based on patient concerns, including poor appearance due to spacing and functional difficulties in eating and speaking, as well as the presenting features, such as facial asymmetry and corresponding malocclusion, delayed or abnormal tooth eruption, and painless swelling or bony expansion. General dental practitioners (GDPs) will often see patients presenting with these multiple symptoms which can be overwhelming. An awareness of the oral and dental features is thus important to make an early diagnosis and to ensure that the patient is given the best advice and guidance early on what is available to address their concerns. This paper provides an overview of the oral and dental features associated with CFD and presents two cases in whom delayed diagnosis resulted in complex treatment needs to address their concerns. Both cases highlight the importance of early diagnosis and the essential role of GDPs in early recognition of the condition to optimise patient care and wellbeing.

## Key points

- Early recognition of the oral and dental features of fibrous dysplasia is important to initiate early management.
- General dental practitioners play an essential role in the early recognition of these features.
- Patients with craniofacial fibrous dysplasia have complex integrated treatment needs which need early recognition, intervention and referral if necessary.

## Introduction

Fibrous dysplasia (FD) is a rare, developmental, benign fibro-osseous disease in which normal bone is replaced with fibrous tissue and structurally weak bone due to a change in normal bone metabolism by somatic gene mutation during embryogenesis.^1^ The clinical symptoms of the disease depend on when the mutation occurs and the level of differentiation of the primary pluripotent cells.^2^ Its incidence is estimated to be 1-in-5,000–10,000^3^ and constitutes 5–7% of all benign bone lesions.^4^^,^^5^ A 2:1 female-to-male ratio has been suggested with manifestation usually in the first few years of life.^6^

FD can be monostotic involving one bone, polyostotic involving multiple bones, or syndromic being associated with Jaffe-Lichtenstein disease, or Mazabraud and McCune-Albright syndromes.^7^ Those with the McCune-Albright syndrome also present with skin pigmentation, with light brown irregularly shaped patches with jagged edges known as ‘café-au-lait spots'. Other features related to endocrine abnormalities include hyperthyroidism, excess cortisol production (Cushing's syndrome), enlarged facial features, hands and feet (acromegaly)^8^ and proptosis (bulging eyes). Hearing loss or optic nerve damage caused by cranial nerve compression may also be seen.^9^ Monostotic FD accounts for 70% of all cases and involves the jaw bones, especially the posterior maxilla, in which painless expansion of the bone occurs, with the term craniofacial fibrous dysplasia (CFD) used to describe cranial bone involvement.^5^ Its prevalence in the craniofacial region ranges from 10–25% in monostotic disease, and up to 90% in polyostotic disease.^10^^,^^11^ The clinical features of FD are influenced by the type and can be isolated to small areas or may involve extensive spread to wider areas. CFD lesions are some of the earliest that can be detected, and the maxilla is affected almost twice as often as the mandible, with the lesions usually expanding during childhood/adolescence and becoming less active in adulthood. Expansion of the alveolar ridge cortices may extend to the hard palate.^12^^,^^13^ Other facial and dental features include facial asymmetry and deformity caused by the bone expansion within the jaw; malocclusion; tooth displacement; dental crowding and spacing; enamel hypoplasia; odontomas and retained primary teeth; diminution of the maxillary sinus; dentine dysplasia; and taurodont pulp chambers.^14^ Patients with CFD may also have a high caries incidence and when present with jaw deformities and café-au-lait lesions, a diagnosis of CFD should be suspected.

Diagnosis of CFD is based on the patient's history and clinical presentation; however, radiographic findings can be variable depending on the extent and severity of the disease. The classical radiographic appearance of the bone is described as ‘ground-glass' or ‘mixed mottled' with interspersed radiolucency and radiopacity and ill-defined lamina dura.^15^ Displacement of the inferior dental canal in the mandible due to abnormal bone formation within the fibrous tissue matrix is also seen.^16^ CFD can be misdiagnosed due to similarities of the clinical and radiographic features with other conditions^17^ (Table 1); however, the use of high-resolution imaging, such as cone beam computed tomography (CBCT) providing 3-dimensional images of the bone structure^18^ and magnetic resonance imaging useful for evaluating bone marrow and soft tissue components, have been invaluable in aiding diagnosis. Recent diagnostic tools, such as molecular and genetic testing with GNAS mutation analysis, are more sensitive^19^ and diagnostic for CFD and diphosphonate bone scanning (scintigraphy) used in conjunction with these other tests can help in determining the extent of the disease and additional sites of activity.^20^

**Table 1:** Differential diagnosis of craniofacial fibrous dysplasia (CFD)

**Differential diagnosis**	**Clinical presentation**	**Radiographic appearance**	**Similarity with CFD**	**Difference with CFD**
Simple bone cyst^41^	Usually asymptomatic and found incidentally Can cause pain or swelling if it expands	Well-defined, radiolucent, solitary lesion with smooth borders and no cortical destruction	Painless swellings Often discovered incidentally	Radiolucent with no internal radiopacities
Ossifying fibroma^42^	Well-defined and slow-growing benign tumour	Expansile lesion with a clearer cortical boundary	Painless bony expansion Mixed radiolucent-radiopaque appearance with cortical thinning and expansion	Well-demarcated and encapsulated
Osseous dysplasia^43^	Non-expansile growth pattern Usually asymptomatic	Radiolucent in early stage which tends to become mixed and eventually radiopaque as it matures	Asymptomatic Often discovered incidentally Ground-glass radiographic appearance	Non-expansile Usually no facial asymmetry
Giant cell tumour^44^	Painful, rapidly-enlarging mass May cause pathological fracture	Purely radiolucency Well-defined lesion Cortical thinning and possible expansion	Facial swelling Radiographically expansile with cortical thinning	Painful, rapidly expanding Radiographically purely radiolucent and multilocular
Aneurysmal bone cyst^45^	Painful and rapidly growing swelling May cause pathological fracture and tenderness	Multiloculated, radiolucent lesion ‘Soap bubble' appearance and cortical thinning or expansion	May result in facial asymmetry Radiographically expansile with cortical thinning or ballooning	Painful, rapidly expanding Cortical perforation may be seen
Paget's disease of bone^46^	Tends to be seen in older adults over the age of 50 More commonly involve multiple bones	Ground-glass/cotton wool appearance	May result in facial asymmetry Ground-glass radiographic appearance	Usually affects older adults Involves multiple bones in the skull
Osteosarcoma^47^	Malignant bone tumour Usually associated with pain, rapid growth and cortical destruction	A ‘sunburst' periosteal reaction and Codman's triangle (triangular area of new bone) appears	Bony swelling Radiographically, poorly-defined, mixed-density lesions with cortical disruption	Painful, fast-growing Causing paresthesia or tooth mobility Radiographically sunburst appearance
Cherubism^48^	Bilateral, symmetrical jaw swelling	Multilocular and expansile radiolucent lesions	Painless swelling of jaw and facial deformity Radiographically – cortical expansion and thinning	Bilateral, symmetrical jaw swelling in children, which regress after puberty
CFD, craniofacial fibrous dysplasia				

Biopsy with a histological analysis, however, remains the gold standard and shows irregularly shaped immature woven bone trabeculae, often described as Chinese characters,^21^ in loosely organised fibrous connective tissue matrix containing spindle-shaped fibroblasts with inactive osteoblasts and a lack of osteoblastic rimming. As the fibrous tissue progressively replaces normal bone, the medullary cavity enlarges, with thinning of the cortical bone, and the fibrous component can be highly cellular or relatively sparse, exhibiting a whorled or storiform pattern.^16^

The most common oral complaints with CFD include poor appearance, discomfort and difficulty in function related to the spacing of the teeth and progressive changes in occlusion as the jaws expand. The primary goal in the management of these patients is to improve aesthetics, function and comfort, with preventive measures underpinning any intervention and the treatment options being influenced by the age at presentation and the extent and behaviour of the disease. Early recognition and diagnosis of the oral features of CFD are essential to minimise protracted courses of intervention and help raise the patient's awareness of the need for prevention to minimise progressive deterioration.

Two patients in whom the early diagnosis of CFD was overlooked resulting in advanced dental challenges necessitating extensive and protracted restorative management are presented.

## Case 1

A 28-year-old white male patient presented complaining of poor appearance and mobility associated with his anterior teeth causing difficulty with chewing.

**Fig. 1:**
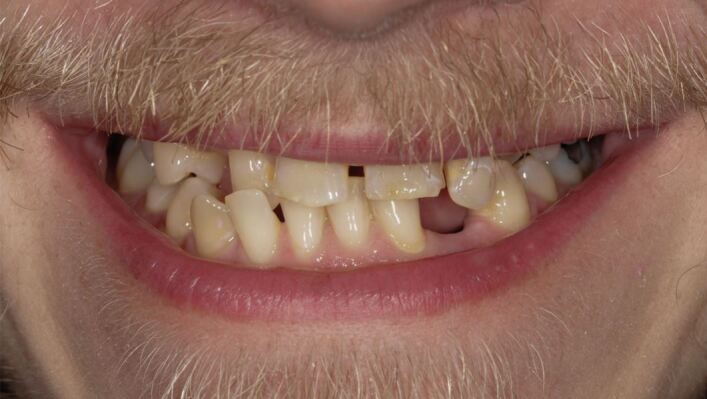
Case 1: extra-oral smiling appearance

A diagnosis of polyostotic CFD affecting both jaws had been made at the age of 15. He had given up smoking one year ago and reported high stress levels. There was a history of orthodontic treatment between 11–16 years of age, with difficulty encountered with tooth movement. He was an irregular dental attender as he was told ‘nothing could be done'. His extra-oral appearance is seen in Figure 1.

**Fig. 2:**
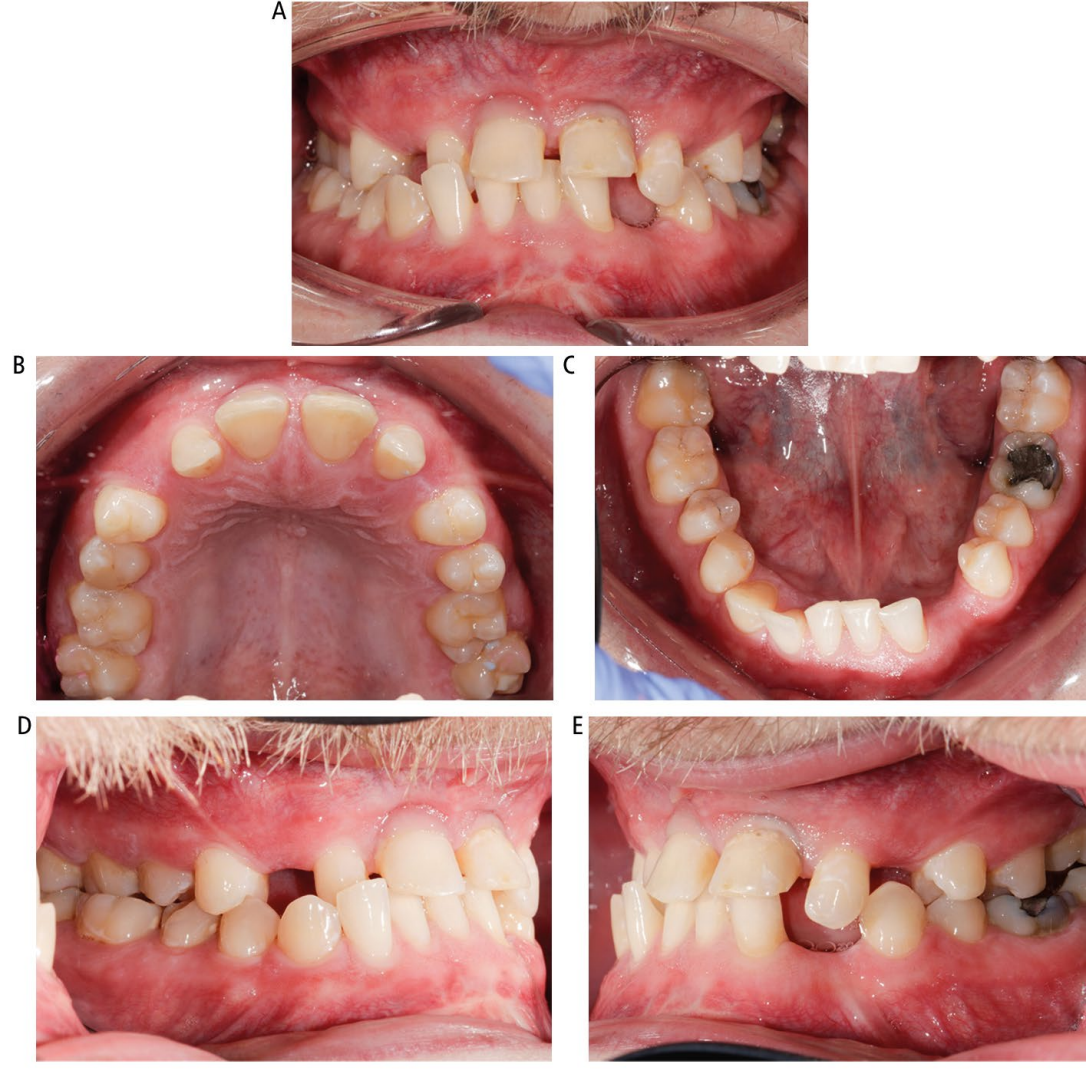
Case 1: intra-oral appearance of the teeth. (A) Cross bite and deep overbite and missing teeth. (B) Expansion of the anterior maxilla with spacing. (C) Expansion of the anterior mandible with spacing and crowding. (D, E) Right and left buccal views

Figure 2 shows the intra-oral presentation. The anterior maxilla and mandible were enlarged with reduced sulcus depth, plus poor oral hygiene with generalised inflammation of the gingival tissues with bleeding and probing depths of 3–5 mm. Teeth 11 and 21 were grade III mobile and 42 was grade II mobile. The gingival tissues were of thin biotype.

**Fig. 3:**
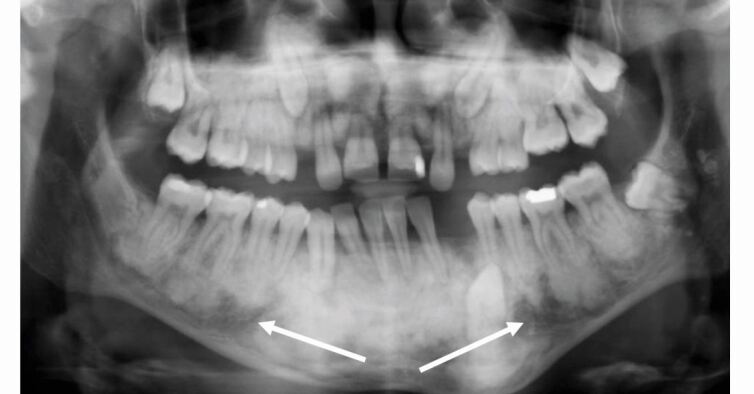
Case 1: orthopantomogram showing the typical ‘ground-glass' and mixed mottled appearance (indicated with arrows), unerupted teeth, taurodont molars, short roots and poorly defined lamina dura and inferior dental canal

**Fig. 4:**
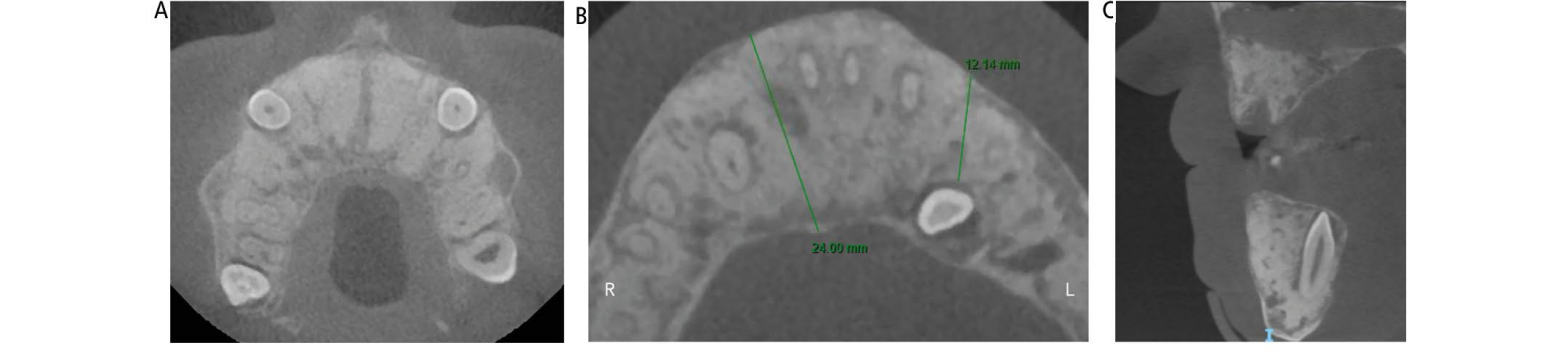
Case 1: cone-beam computed tomography scan. (A, B) Axial views of the maxilla and mandible showing the bone expansion and the position of the impacted canine teeth. (C) Sagittal view of the anterior maxilla and mandible showing the bone expansion and the position of the impacted canine tooth

Figure 3 and Figure 4 show the radiographic appearance and CBCT of the jaws, confirming the bony appearance and expansion.

Diagnoses of localised periodontitis stage III, grade C, currently unstable with the smoking history and stress as the risk factors, impacted teeth and CFD causing the spacing and malocclusion of the dentition, were made. The prognosis of the upper incisor teeth was poor. The occlusion and the underlying CFD, associated with the unpredictability in its response to extraction and dental implant surgery, posed challenges in addressing his main concerns, complicated by his expectations, attitude and compliance.

The dental management was phased and the primary challenges of treatment, including the unpredictability of the outcome, the risk of bleeding, and the issues with quality of the bone and its influence on the provision of dental implants, were discussed with the patient. Further engagement with the patient focused on ensuring that he understood these challenges and his commitment and attitude to the treatment needed to address his concerns. Treatment was executed as follows.

### Initial phase

The *S3 Treatment Guidelines for Periodontitis*^22^ were followed to manage the periodontal issues. A diagnostic wax-up to show the feasibility of space closure in the anterior region on loss of 12 and 11 and 21 and 22 was shown to the patient. A decision was made to proceed with a transitional denture due to the uncertainty of the healing response to the extraction due to the underlying CFD. At review, patient compliance had improved with resolution of the periodontal problems. The patient was reminded of the previously discussed risks and challenges and consented to proceed with the next step.

### Interim phase

**Fig. 5:**
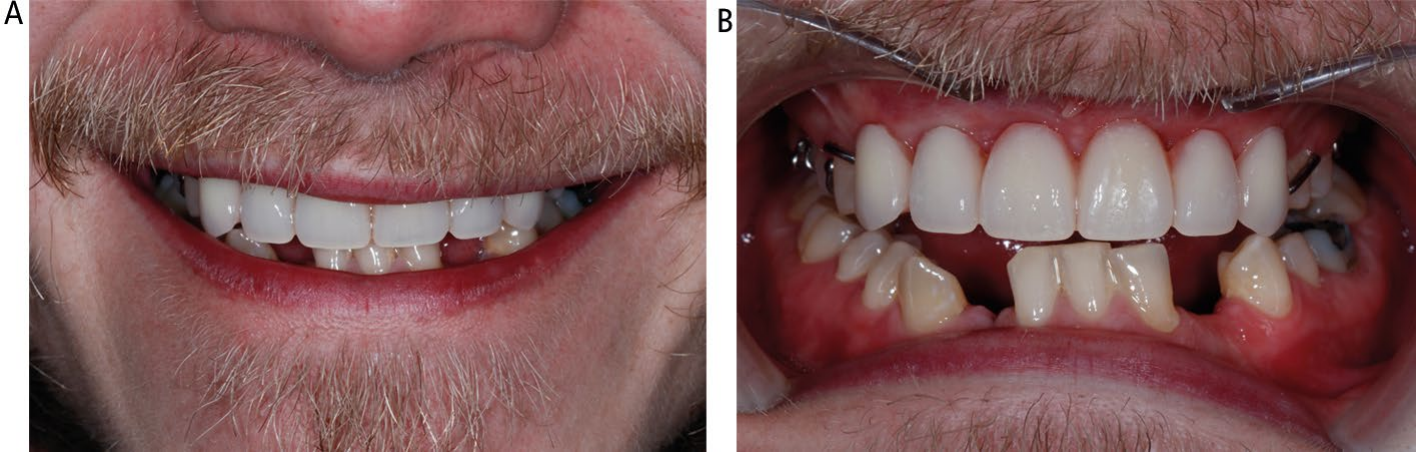
Case 1: improved appearance following fit of the immediate partial denture. (A) Smiling. (B) Retracted

An immediate tooth-supported upper partial denture with no anterior flange was fitted following extraction of the teeth (Fig. 5) and 42 was also extracted and ongoing maintenance was implemented. Healing was uneventful at one week and the denture was relined with tissue conditioner and the lower spaces were maintained. He was delighted with the improved comfort and enhanced aesthetic outcome. The definitive tooth replacement options were revisited, and dental implants were considered as the fixed option. The associated risks, including the uncertainty with vascularity and increased risk of bleeding, the quality of bone due to CFD, the possible impact on the wound healing following surgery and the issues with osseointegration of the implants,^12^ were discussed, and detailed planning was undertaken, taking into consideration the management of the unerupted canines.

### Definitive phase

**Fig. 6:**
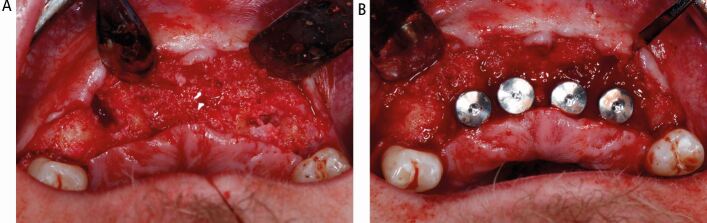
Case 1: at first stage implant surgery. (A) Fibrous appearance of the alveolar bone with expansion. (B) Implant fixtures *in situ*

**Fig. 7:**
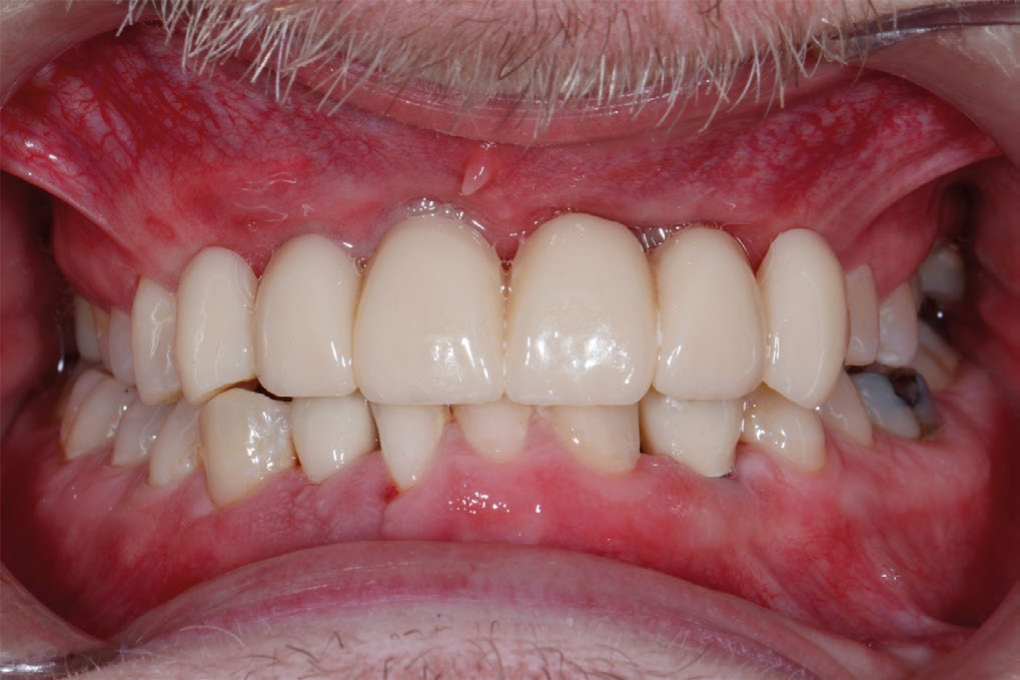
Case 1: upper implant-supported bridge and lower right and left implant-supported crowns *in situ*

**Fig. 8:**
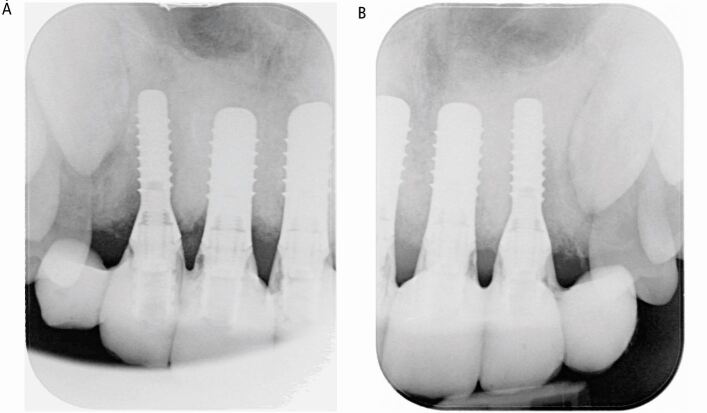
(A, B) Case 1: peri-apical radiographs showing the implants placed in the anterior maxilla

Six months later, restoratively driven implant placement with four implants in the maxilla and two in the mandible at an insertion torque of 35 Ncm was undertaken (Fig. 6). Despite the fibrous nature of the bone, high primary stability was achieved and healing one week later was uneventful. A provisional bridge was fitted five months after the surgery in the maxilla and crowns fitted in the mandible (Fig. 7). The radiographs taken post-fit (Fig. 8) showed the ground-glass bone appearance. The radiolucency seen on the distal of the lateral incisor implants could be the healing response of the CFD as clinically there was no pocketing, bleeding or inflammation noted around the implants. The patient was delighted with the outcome.

The patient has been kept under regular follow-up to ensure his compliance is maintained. His attitude to dental treatment has improved and the gingival tissues around the implants continue to remain stable despite the lack of keratinised tissue which is unlikely to be related to the CFD. The patient has requested a break from treatment and the provision of the final prosthesis has thus been delayed. When he is ready to embark on the final stage of treatment, the site will be reassessed and the need to increase the keratinised gingival tissue will be considered.

Table 2 summarises the key challenges encountered in the management of this case and outcomes achieved.

**Table 2:** Key challenges and outcomes in Case 1

**Challenge**	**Reasons**	**Outcomes**
Patient's attitude to treatment and lack of engagement	Lack of appropriate information at the outset: Patient being told that nothing could be done and he had to accept the appearance and difficulty in chewing, resulted in non-compliance, poor behaviour and engagement	Supportive discussion, dialogue to improve understanding about his role and how he could help address his concerns
Patient's expectations	Refused to accept that a fixed prosthesis would be difficult	Phased treatment plan highlighting the importance of sequenced treatment to mitigate risks
Multiple risk factors – stress, smoking and unstable periodontal disease	Lack of understanding and awareness of the effect of risk factors on his oral health although he understood his CFD well but not the oral aspects	Behavioural change and engagement into the process with excellent compliance once patient gained understanding of the treatment process; drastic change noted after fitting of the denture
CFD and the associated challenges with predictability of treatment options	Mixed activity of CFD with increased risk of bleeding; challenges with fixed tooth replacement options	Multiple discussions with patient-centred planning to engage patient in the decision making while ensuring realism
Multiple impacted teeth	Influenced the choice of treatment options	Careful and pragmatic planning with no issues encountered
Osseointegration of dental implants	Lack of bone and fibrous stroma complicating osseointegration; poor evidence base on treatment outcomes	Successful outcome of implant treatment with risk-assessed planning approach using his interim denture to achieve the aesthetics
CFD, craniofacial fibrous dysplasia. This case of a young patient highlights the impact of lack of appropriate information given at the outset about oral health can have a domino effect on management, behaviour and attitude to care. The successful outcome achieved emphasises the role of clear communication in such cases to manage expectations overcoming the behavioural challenges while maintaining realism. The importance of phasing in multidisciplinary treatment is also highlighted, as it enables careful, stepwise management of each contributing condition, managing risks and stabilising them before proceeding to the next. The improved patient attitude, expectations and compliance early in the journey was fundamental to balancing the surgical risks associated with implant provision in this patient with unknown CFD status.		

## Case 2

A 78-year-old Afro-Caribbean female patient presented complaining of poor appearance due to a failing bridge and loose teeth causing difficulty with chewing.

A diagnosis of monostotic CFD affecting the right maxilla managed with three-monthly intravenous infusions of pamidronate was made in 2015. Hypertension, osteoarthritis, schizophrenia, and well-controlled type II diabetes mellitus were noted. The patient reported to be a non-smoker and an irregular dental attender.

**Fig. 9:**
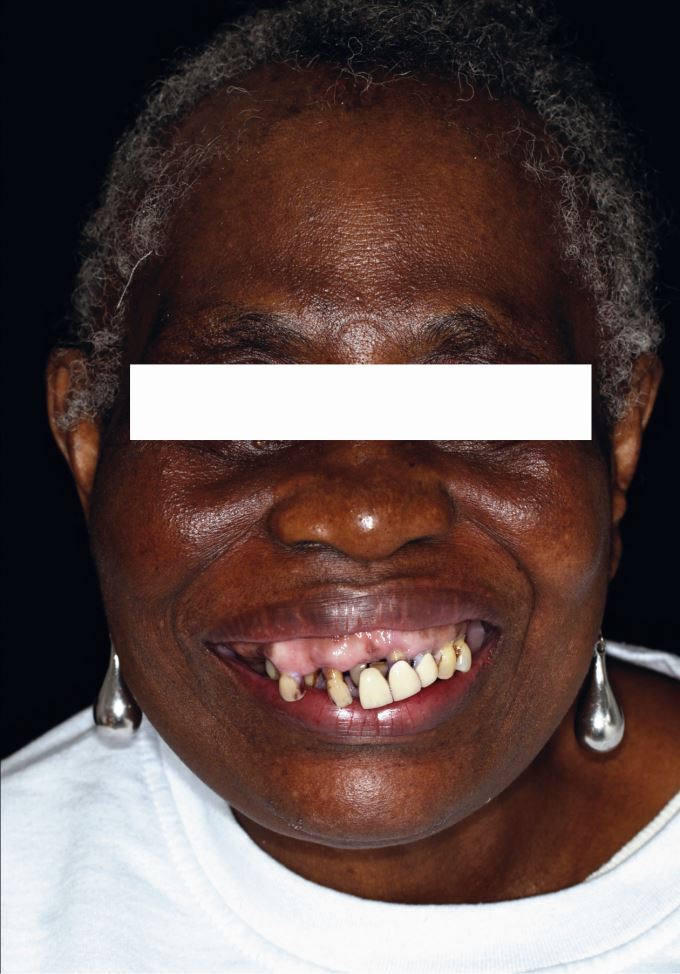
Case 2: Extra-oral appearance showing facial asymmetry, high smile line, and cant of the maxillary occlusal plane

The extra-oral appearance with a severe cant on the right side is seen in Figure 9.

**Fig. 10:**
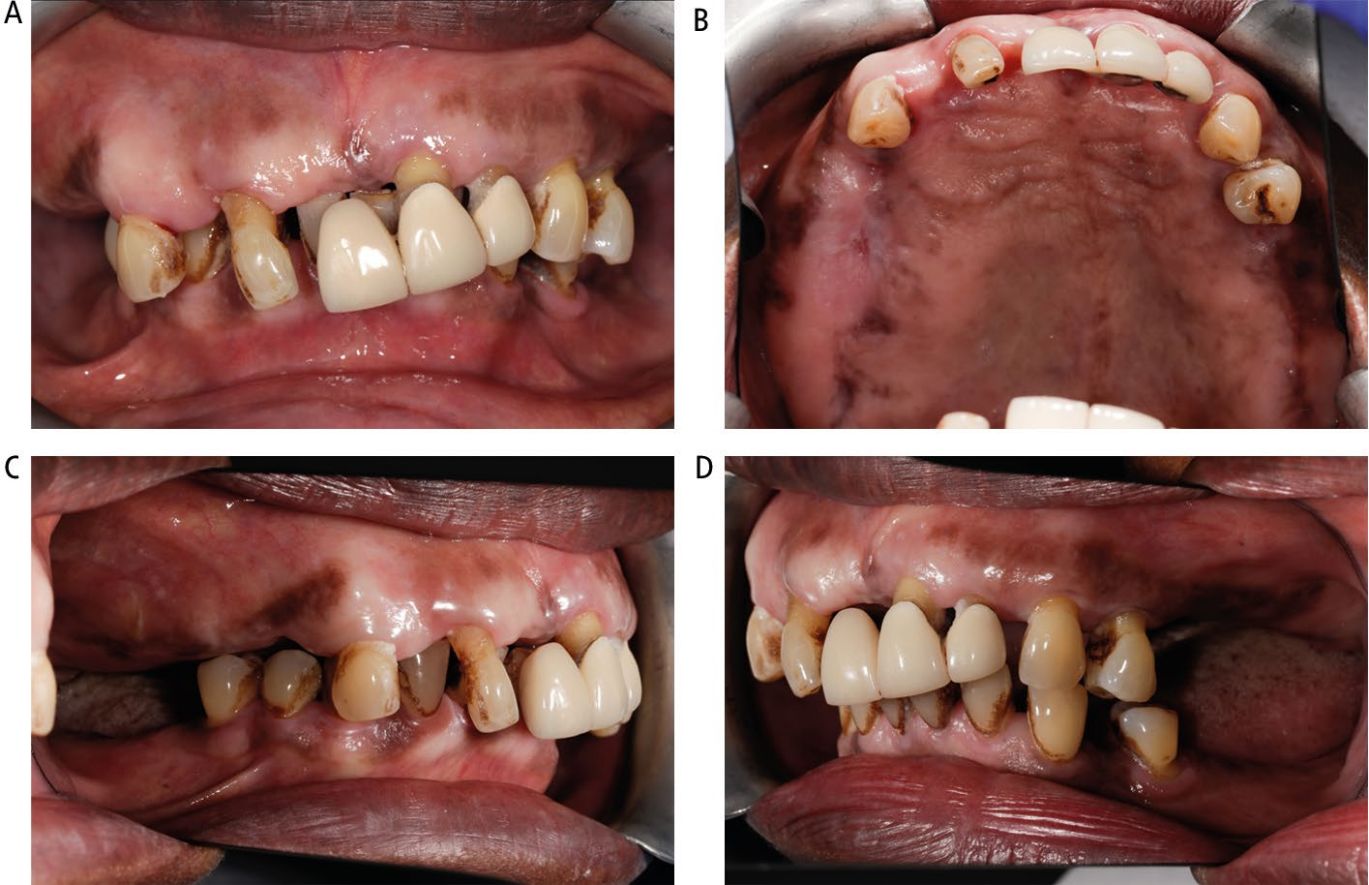
Case 2: intra-oral views. (A) Right cant with teeth in occlusion. (B) Palatal view showing the bony expansion of the right maxilla. (C, D) Right and left lateral views showing the lack of interocclusal space on the right side

The intra-oral appearance is shown in Figure 10, with the expansion of the right maxillary alveolus causing the occlusal cant. The oral hygiene was poor, with inflamed gingival tissues with bleeding and probing depths of up to 7 mm. The upper anterior bridge was failing with extrusion of the grade III incisor teeth and missing molars with a traumatic overbite.

**Fig. 11:**
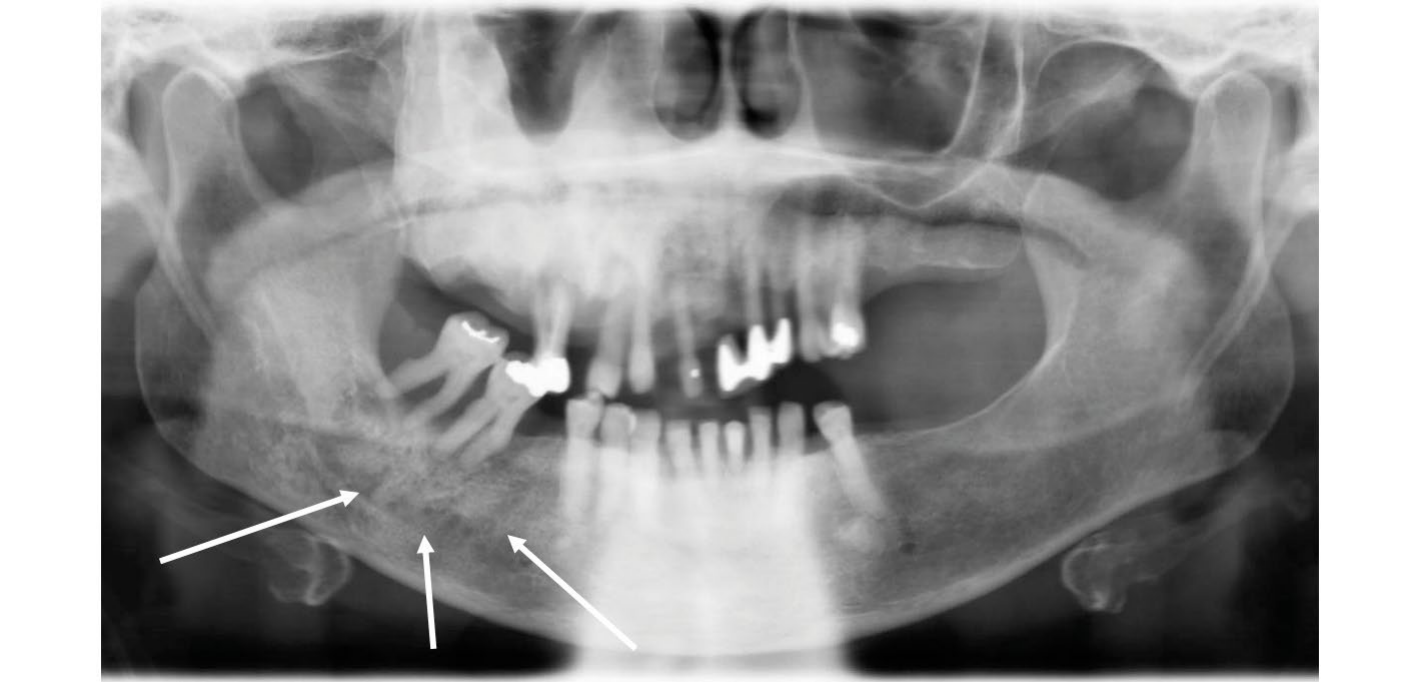
Case 2: orthopantomogram showing the ground-glass and mottled appearance of the alveolar bone (indicated with arrows) and generalised horizontal bone loss

The ‘ground-glass' radiographic appearance of the bone in the right maxilla and mottled appearance in the lower right mandible are evident in Figure 11.

Diagnoses of generalised periodontitis stage IV, grade C, currently unstable with controlled diabetes, CFD, acquired tooth loss and failing upper anterior bridge were made. The prognosis of the upper teeth and the lower right molar teeth was poor and that of the remaining teeth was questionable.

The dental management was complicated by the differential size of the maxilla due to the bony expansion, the maxillomandibular relationship, deep overbite, intravenous drug infusions and patient compliance. To correct the malocclusion and facial asymmetry, the options of surgical bone recontouring and orthognathic surgery^23^ were considered; however, due to the risks with the intravenous drug infusion, risks of recurrence following surgery^24^ and the patient's intolerance to extensive surgical procedures, these were discarded. The patient's focus was the maxillary teeth only and treatment was thus planned with a phased approach aimed at the upper jaw keeping status quo in the mandible. The associated challenges with the failing teeth in line with her expectations and compliance was discussed and consent was obtained for treatment executed as follows.

### Initial phase

**Fig. 12:**
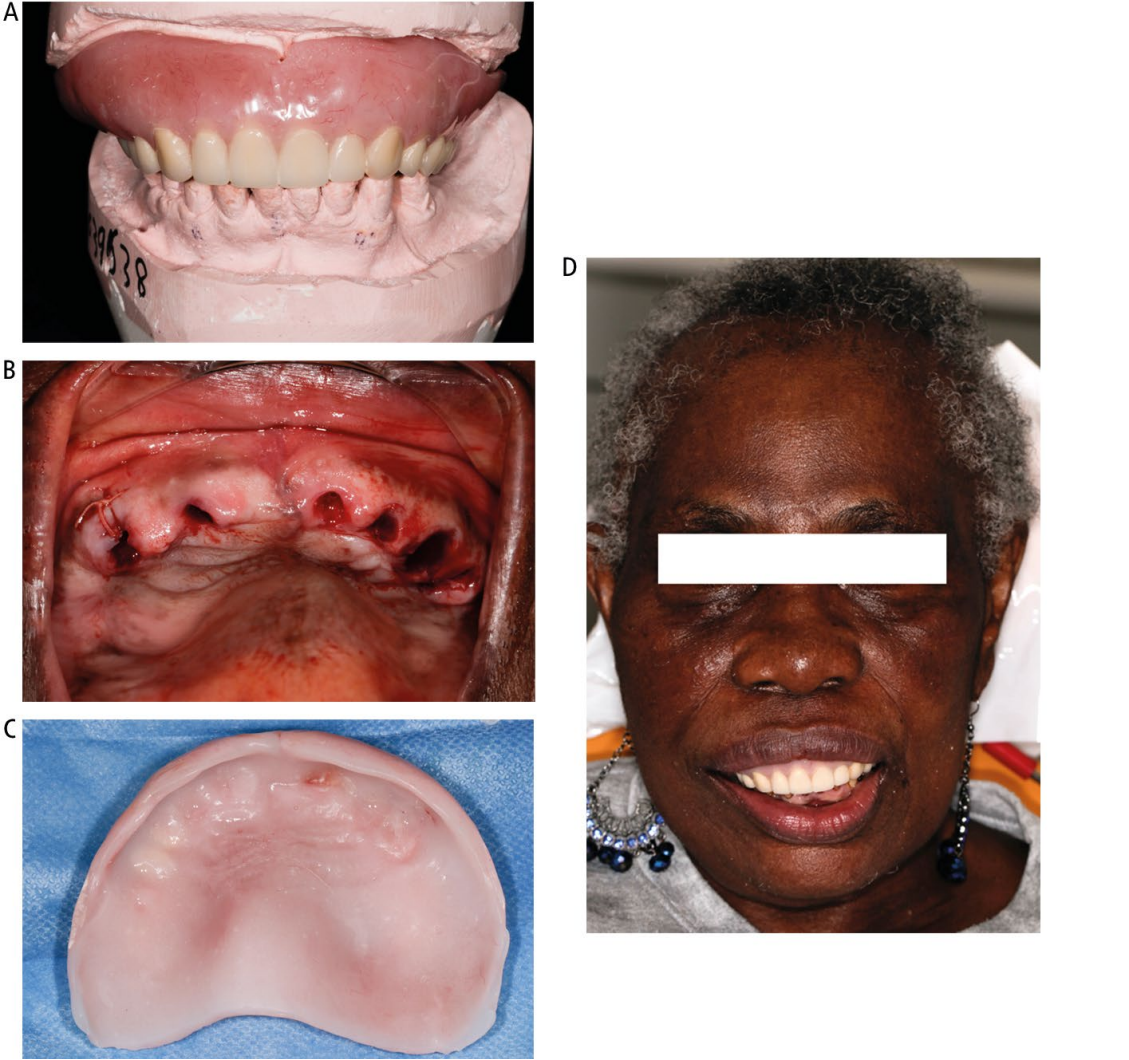
Case 2. (A) Immediate maxillary complete denture on articulated working models. (B) Maxillary arch clearance. (C) The immediate maxillary denture relined with temporary soft denture liner. (D) Appearance following fit of the denture fitting of the immediate maxillary denture

The *S3 Treatment Guidelines for Periodontitis*^22^ were followed to manage the periodontal disease and her role highlighted while planning removal of all the upper teeth and their replacement with an immediate upper denture in conjunction with the medical practitioner and the laboratory technician. The latter was essential to establish how much the cant could be corrected (Fig. 12).

### Interim phase

The upper teeth were removed and the denture was fitted. The intravenous drug infusion was stopped for three months after the extractions. One week later, healing was uneventful and the denture was relined with tissue conditioner. The patient was delighted with the outcome.

### Definitive phase

**Fig. 13:**
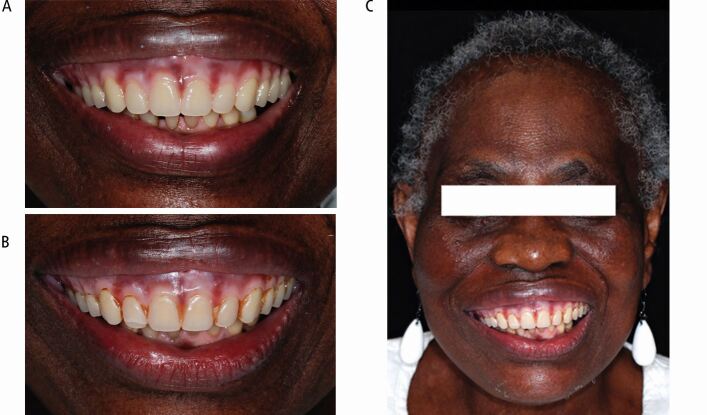
Case 2: definitive maxillary complete denture. (A) Fit appointment. (B) Smile at review 15 months. (C) Facial view

The definitive denture, constructed using copy denture technique, was fitted and the patient was reviewed for 18 months (Fig. 13). The patient remained compliant and showed a new level of confidence with the outcome.

Table 3 summarises the key challenges encountered in the management of this case and outcomes achieved.

**Table 3:** Key challenges and outcomes in Case 2

**Challenge**	**Reasons**	**Outcomes**
Patient's attitude to treatment and poor compliance	Caused by being told that nothing could be done for her other than surgery and she had to accept that she would have no teeth eventually	Supportive discussion, highlighting the challenges and options
Medical history which included hypertension, osteoarthritis, schizophrenia and three-monthly intravenous pamidronate infusions	IV infusions placed greater risks with possible bone necrosis	Patient did not want surgical intervention which carried higher risks; non-surgical management to address her concerns using a team approach for planning
Severe maxillary cant; unstable periodontal disease with multiple failing teeth; patient focus was the maxilla only	Severity of periodontal disease rendering prognosis of teeth poor; challenge with addressing this with the severe cant in the absence of surgery	Careful planning engaging the laboratory technician using a stepwise approach with wax up before any invasive intervention undertaken
Failing dentition with poorly prognosed teeth; risk of bone necrosis due to intravenous medication	Unstable periodontal disease due to lack of early guidance and information	Successful extractions with no complications managed with the medical team and provision of a carefully planned denture
Monostotic CFD with a possible slow-growing lesion influencing the predictability of treatment	Severe cant which patient felt was worsening and affecting appearance and function	Keeping treatment simple and relatively non-invasive resulted in an improved outcome which the patient was happy with
CFD, craniofacial fibrous dysplasia. This patient was an older patient with low expectations and given the severity of the issues and the challenges, adaptation to treatment due to age could be a potential issue. However, in this case, although 78, she was biologically young and her focus was to look better and be able to eat without pain, both of which were significant factors that facilitated her engagement into the treatment journey. Despite the complexity of the medical history (severe maxillary cant and treatment being complicated by the surgical risks), the patient engaged with the agreed treatment plan which was patient-centred. The successful outcome of simple treatment provided was achieved by coordinated engagement not just with the patient but also with her medical team and the dental laboratory technician. The successful outcome was not just about the teeth but also about the impact on this patient who had demonstrated renewed confidence highlighting the important role clinicians play in early management of dental issues to avoid long-term psychological issues.		

## Discussion

Both cases had a diagnosis of CFD but posed different challenges in their dental management; however, both were complicated by the poor attitude/compliance due to erroneous information that ‘nothing could be done' given to them. The effect of oral health on patients' quality of life has been reported^25^ and this was evident in both patients in whom as soon as the pain was resolved and the appearance improved, there was an increased confidence and positivity seen towards their dental and oral health.

The clinical presentation of the first case is in line with the features published for polyostotic CFD.^10^ The key challenge in this case was the patient's attitude and behaviour. A phased treatment approach allowed for the exploration of these issues while simultaneously assessing the stability of the CFD and evaluating the patient's expectations. The decision to opt for implant treatment was influenced by the patient's age and his desire for a fixed option, and was only undertaken after careful evaluation of the healing and post-extraction bone quality. It is reported that endo-osseous implants in patients with CFD are at a higher risk of failure due to a poorer bone quality, rate of bone healing and osseointegration.^26^ In our case, there was good vascularity and healing which was evident following the extraction, and high primary stability of the implants had been obtained at high insertion torque, thus giving the confidence of a good outcome. A five-year successful outcome has been reported for implants in patients with CFD^27^ and others have reported similar success with complete re-ossification of the bone and integration of implants.^28^^,^^29^^,^^30^ The risk of failure is reported to be reduced if the implants are placed after skeletal maturity when the growth of CFD lesions have diminished.^28^ Implant outcomes in both CFD bone and normal bone have been reported to be comparable;^29^ although, one study with a full arch maxillary rehabilitation reported a failure of one implant due to peri-implantitis after six years.^31^ One study reported successful osseointegration of an implant placed in the maxilla subsequent to surgical CFD lesion removal with histologically confirmed healing, with one-year peri-implant bone level stability.^32^ Another study reported successful surgical recontouring of CFD that developed around a dental implant in the maxilla, with preservation of peri-implant soft and hard tissues over a six-month follow-up.^33^ In the case presented, the implants have been *in situ* for just over three years with no complications. This outcome was underpinned by integrated treatment planning with careful consideration of the bone quantity and quality using a phased treatment approach with atraumatic surgical techniques used for implant placement. In this case orthodontic treatment had been attempted; however, this was unsuccessful due to lack of tooth movement, which is contrary to published evidence which states that orthodontic tooth movement can be undertaken in CFD cases; although, a high tendency of relapse has been reported.^11^ The root shortening associated with the central incisor teeth could be due to the orthodontic treatment and subsequent tooth mobility related to the unstable periodontal disease and deep overbite. The quality of the gingival tissues was difficult to establish at the outset; however, once the periodontal health was stabilised, thin gingival biotype was noted. The remodelling changes post-extraction may have been contributory to the change in the gingival tissues and the lack of keratinised tissue is often reported around dental implants. In this case, this is unlikely to be related to the CFD; however, before the definitive implant provision, creating a band of keratinised tissues should be considered to minimise the risk of future peri-implant disease.^34^ The effect of CFD on keratinised tissues and periodontal disease remains unanswered with little to no evidence published. In this case, the cause of the periodontal disease was primarily his plaque control with his smoking history as the risk factor, and if the CFD had been a major issue, the outcome to the debridement would not have been successful. The treatment executed up to the provision of the denture is straightforward and falls within the remit of a general dental practitioner (GDP), who needs to be mindful of the challenges in patient management due to the stigma. It is important to recognise the benefits of early recognition, stage the treatment to address concerns promptly, and refer the patient early when necessary.

The second case presented the classical features of monostotic CFD, including a slow-growing and painless unilateral expansion of the posterior maxilla,^13^^,^^35^ with a late diagnosis made in the second decade of life noted as an incidental radiographic finding.^28^^,^^36^ The slow-growing nature of the lesion was confirmed in this case where the progressive increase in the spacing between the teeth had prompted her to seek help with the secondary periodontal issues that had led to pain. The intravenous infusion complicated the provision of the dental management due to the increased risk of the medication-related osteonecrosis of jaw (MRONJ).^37^ The case highlights the importance of close integrated working with medical practitioners to manage the risks and also emphasises the importance of controlling primary diseases, which in her case had exacerbated the ongoing discomfort and pain from the teeth.^37^^,^^38^ Dental practitioners should be aware of the Scottish Dental Clinical Effectiveness Programme guidelines which provide clear guidance on how to manage patients taking anti-resorptive drugs regarding preventive advice, understanding the risks of extraction, and undertaking close clinical and radiographic monitoring.^39^ While surgical correction of the excess bone could have been considered, due to the risk of MRONJ, this was deemed inappropriate. It has been reported that even after surgical correction, problems with denture retention and stability can be seen due to adaptability.^23^^,^^40^ The denture offered the flexibility of correcting the cant and is a more cost-effective alternative, especially in the presence of uncertainty related to the CFD activity. This patient was keen on staying with a denture and such patients can be managed effectively by GDPs; however, where complexity is driven by the presence of facial deformities, lack of sufficient restorative space, asymmetries and severe malocclusion with unstable jaw dimensions, a referral to a specialist should be considered.

Both cases highlight the importance of early recognition and intervention by GDPs. While the tooth drifting and mobility could be secondary to the CFD, the unstable periodontal disease, which is preventable, had led to the exacerbation and poor prognosis of the teeth, and early intervention could have minimised this risk. This is essential to ensure that patients are being given the appropriate guidance and advice at the outset and as most of the population are first encountered by the GDPs, they play a key role in early diagnosis and/or referral. The key areas that GDPs should be mindful of when seeing such patients are:

- Listening and hearing the patient's complaints, especially if the complaint is of poor appearance due to spacing or tooth movement or unusual facial asymmetry. Further questions should be asked about duration and timing and if it is worsening
- Patients complaining about ‘pigmentation'. Those with appearance of café-au-lait spots should be questioned further about their medical history and the syndromic CFD suspected
- Clinical examination and full mouth assessment should be undertaken to exclude other causes of tooth movement, e.g., periodontal disease, apical infection. Preventive care should be initiated, and the patient should be reassessed to evaluate the next steps in the event of no improvement or change
- Ensure communication with the patient is clear, informative and relevant, providing guidance on the treatment progress, and highlighting their role
- Discuss treatment options ensuring the patient understands the limitations
- When feeling overwhelmed, consider referral but initiate primary dental disease management with patient engagement.

GDPs, as the gatekeepers to patient care, play an essential role in the ongoing management of patients for their day-to-day oral health needs and even for patients who are referred.^13^^,^^35^

**Fig. 14:**
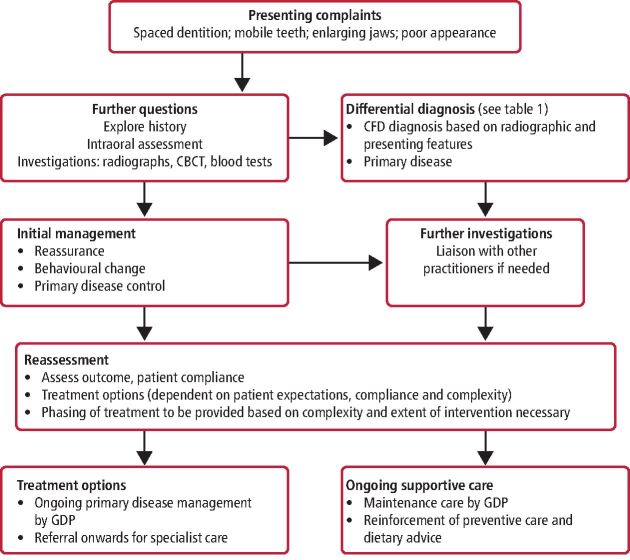
Diagnostic and treatment pathway for patients with suspected craniofacial fibrous dysplasia

Figure 14 provides an overview of the patient journey in the management of patients with CFD bringing together the concepts covered in the cases presented.

## Conclusion

Craniofacial fibrous dysplasia is a rare condition that can affect patients who present to GDPs. In these patients, preventive care and early intervention will help towards maintaining the dentition and oral health; however, this must be an integrated and collaborative approach, with the patients at the core of the management. The cases presented have highlighted the importance of early intervention while emphasising the importance of a patient-centred individualised approach to managing their expectations and compliance. In both cases, the planning was undertaken with patient engagement and with a joint decision-making approach, taking into account factors such as the patient's age and skeletal maturity, as well as their compliance and the location, severity and behaviour of the lesions. All these have to be considered when planning a patient's management, recognising that dental procedures are safe to undertake in patients with CFD.

GDPs are proficient in managing a wide spectrum of dental diseases and conditions which should include CFD. On the other hand, some cases of CFD may exhibit intricacies and complex treatment needs and may need early referral management to a specialist or tertiary care. Even in these cases, the GDPs play a pivotal role, not just in the management of the primary disease stabilisation, but also in the provision of maintenance and supportive care needed by the patients afterwards to maintain a successful outcome.
